# Predictive factors of endometriosis progression into ovarian cancer

**DOI:** 10.1186/s13048-021-00940-8

**Published:** 2022-01-10

**Authors:** Ján Varga, Alžbeta Reviczká, Hedviga Háková, Peter Švajdler, Miroslava Rabajdová, Alexander Ostró

**Affiliations:** 1 Department of Gynaecology and Obstetrics, Faculty of Medicine, P.J. Šafárik University and L. Pasteur University Hospital, Rastislavova 43 Street, 041 90 Košice, Slovakia; 2 Frauenklinik, DONAUISAR Klinikum Deggendorf, Deggendorf, Germany; 3 Department of Laboratory Medicine, subdivision of Medical Genetics L. Pasteur University Hospital, Košice, Slovakia; 4 CYTOPATHOS, s.r.o, Bratislava, Slovakia; 5 Department of Medical and Clinical Biochemistry, Faculty of Medicine, P. J. Šafárik University, Košice, Slovakia

**Keywords:** Endometriosis, Endometriosis associated ovarian cancer, Conventional cytogenetics, Atypical endometriosis, CTNNB1, HIF1A

## Abstract

**Background:**

In recent years, the endometriosis has overcome a noteworthy renaissance in the recognition of its potential. In certain patients, a demonstrable malignant progression of ectopic foci leading to development of ovarian cancer is seen. The knowledge of endometriosis overthrow background into endometriosis associated ovarian cancer is of paramount importance for selection of patients at risk. The goal of the presented study was to review a malignant potential of the endometriosis and to specify predictive factors of endometriosis progression into ovarian cancer. Altogether 189 patients were included in the study. Conventional cytogenetics as well as measurement of transcriptional activity of *CTNNB1* (β-catenin) and *HIF1A* (HIF1-α) genes were prospectively studied in 60 endometriosis patients and 50 control group patients. The retrospective histopathological analysis was performed in 19 endometriosis associated ovarian cancer patients and 60 patients with histologically confirmed endometriosis.

**Results:**

Five endometriosis patients showed a deviation from normal cytogenetics finding without affecting of their phenotype. In 6 cases of endometriosis associated ovarian cancer ectopic endometrium was not confirmed. The remaining 13 cases demonstrated either benign or atypical endometriosis or even structures of borderline carcinoma. Atypical endometriosis was histologically confirmed in 20% of 60 endometriosis patients. Determination of gene expression (*CTNNB1, HIF1A*) formed two subgroups. Transcriptionally incipient endometriosis subgroup with insignificant genes expression compared to control group. In transcriptionally evident endometriosis subgroup were genes expressions significantly higher compared to control group (*p* < 0.01) as well as transcriptionally incipient endometriosis subgroup (*p* < 0.05).

**Conclusions:**

Significant structural abnormalities of chromosomes are not included in genetic rigging of endometriosis patients. Atypical endometriosis represents a histopathologically detectable intermediate of endometriosis progression. Determination of genes expression *CTNNB1* and *HIF1A* helps to allocate risk patients with endometriosis where more precise management is needed.

## Background

In recent years, the endometriosis has overcome a noteworthy renaissance in the recognition of its potential. Today, it is understood as a clinically complex syndrome characterized by chronic hormone-dependent inflammation with notable proliferative potential. About 10% of the reproductive age women suffer from this disease and a variety of clinical symptoms are known. In certain patients, a demonstrable malignant progression of ectopic foci leading to development of ovarian cancer (OC) is seen.

The large histological variability of OC presupposes more tissues as an initial structure for the process of ovarian carcinogenesis [1]. In the ovary itself, ovarian surface epithelium (OSE) constitutes the main structure which either accumulates mutations or undergoes metaplasia to the müllerian epithelium. In both cases, the process of carcinogenesis takes place in cortical inclusion cyst (CIC) after incorporation of pathological tissue. Another source of tissue for OC represents an ectopic müllerian epithelium transported and incorporated into CIC. This is the case of endosalpingiosis as a potential source of serous borderline ovarian tumour [2, 3]. The fallopian tube plays an important role in process of OC development. Precursors, such as serous tubal intraepithelial carcinoma (STIC) or papillary tubal hyperplasia (PTH) can locally progress or more often are adhered to carcinogenesis more favourable environment of OSE. Finally, retrograde endometrial reflux adhered to the OSE is involved in the development of some carcinomas, type I (Table 1). Inflamed stroma and mutations containing ectopic endometrium have better conditions to progress in more propitious microenvironment of the ovary [4]. This fact confirmed also finding that deep infiltrating endometriosis although containing the same changes in stroma and epithelium progress into carcinoma sporadically [5].

**Table 1:** Process of ovarian carcinogenesis

Initial structure	Biological process	Final structure
OSE	Mutation + incorporation into CIC	CIC
OSE	Metaplasia + incorporation into CIC	Müllerian CIC
Ectopic müllerian epithelium	Transport to the ovary	Müllerian CIC
Endosalpingiosis	Transport to the ovary	Serous borderline ovarian tumor
Fallopian tube epithelium	Transport to the ovary	Müllerian CIC
STIC	Local progression	Primary fallopian tube carcinoma
STIC	Transport to the ovary	HGSOC
PTH	Transport to the ovary	LGSOC
Endometriosis	Retrograde reflux	EOC, CCOC

The connections of biologically different tissues have been already confirmed as *locus minoris resistentiae* to the carcinogenesis initiation in human body (gastro-esophageal or ano-rectal junction). The connection of the fallopian tube and the ovary, tubo-ovarian junction as well as the process of ovulation in its vicinity represent an impeccable interplay to start the formation of pathogenic clone of cells.

### Endometriosis associated ovarian cancer

The term endometriosis associated ovarian cancer (EAOC) has didactic dimension and in clinical practice there assign mainly endometrioid ovarian cancer (EOC) and clear cell ovarian cancer (CCOC). Not every EAOC has histologically proven endometriosis in its structure. Based on this findings, in can be assumed that endometriosis is a precursor of only certain EAOC. Malignant reversal of endometriosis is an uncommon event, counting for less than 1%. Question which endometriotic lesion tends to progress into carcinoma remains unanswered.

Common features typical for endometriosis and cancer cells, i.e. to evade apoptosis, ability of stem cells as well as angiogenic potential were described. Haemolysis, a process typical for ectopic endometrium is strongly associated with oxidation. The compounds included in process are extracellular free haemoglobin, heme, and iron derivatives. These components were abundantly proven in peritoneal fluid as well as in fluid of endometriomas during menstruation [6]. Oxidation processes in endometriosis deposits result in the accumulation of DNA mutations that with the help of immune system lead either to cell death or formation of pathogenic clone of cells [7].

Despite the development of molecular genetic techniques, classical – conventional cytogenetics is an essential part of both, basic and advanced genetic testing. By the examination the numerical as well as structural chromosomal aberrations are detectable. Based on literature, cytogenetic examinations of endometriosis patients brought often discrepant results.

Based on histopathological criteria a benign (typical) and atypical endometriosis (AE) can be defined, with AE significantly associated in EAOC [8]. Two degrees of atypia were in tissue of AE described. Cellular atypia (cytological) indicating a changes in epithelial layer including hyperchromasia and pleomorphism. Structural atypia (hyperplasia) represent a hyperplastic changes similar to eutopic endometrium (simplex or complex hyperplasia with or without cellular atypia) [9]. Most studies refer AE as a tissue containing both, cellular and structural atypia. However cytological atypia are mostly in cancer free patients seen, whereas structural atypia are found particularly in OC patients [10].

Different clinical potential of both atypia confirmed the studies of COX-2, Ki-67 and BAF250a. Immunohistochemical COX-2 positivity was significantly higher in benign endometriosis (BE) comparing to AE. The same phenomenon was seen when compared cytological versus structural AE. Four time higher COX-2 expression was in cytological AE confirmed. This conclusions predict cytological AE to reactive changes. Ki-67 positivity was significantly higher in tissue of structural atypia confirming greater proliferative potential when compared to cytological atypia. Comparable results were also in case of AE versus BE seen. *ARID1A* mutation phenotypically leading to decrease in protein BAF250a represents an initial genetic change of endometriosis overthrow. BAF250a decrease was confirmed in both, OC as well as AE. When compare structural atypia versus cytological atypia of AE, lower BAF250a expressions were in patients with structural atypia seen [10].

Several studies have confirmed the presence of both, BE and AE in EAOC patients [10–12]. The tissue of EAOC can be also without endometriosis, or with gradual transition from BE to AE and borderline carcinoma (BOC). In case of AE the structural atypia are more common [10]. Currently accepted histopathological criteria for AE include features of eosinophilic cytoplasm, large hyperchromatin or pale nuclei with moderate to marked pleomorphism, increased nucleus to cytoplasm ratio and cell aggregation.

Catenin beta 1 (CTNNB1) called also β-catenin represents a protein encoded by the gene *CTNNB1.* It is involved in the process of carcinogenesis of many cancers. Mainly in the regulation of gene transcription as well as cell adhesion. Activation of the Wnt signaling pathway accompanied by *CTNNB1* mutation induces a process of ambient fibrotization [13]. In addition, the potentiation of proliferation as well as an increase in implantation ability or invasion is recorded [14]. *CTNNB1* mutation was detected in many cancers including ovarian [15] or endometrial type as well as in endometriosis [16]. The regulation of β-catenin is provided by two processes. It is by β-catenin destructive complex and partially also by adenomatous polyposis coli (APC) protein which is encoded by tumor suppressor *APC* gene. The *APC* gene mutation is in several cancer seen, significantly in colorectal carcinoma.

Both, *CTNNB1* mutation as well as Wnt signaling pathway activation was in ectopic endometrium confirmed. The strong association between Wnt pathway activation and fibrosis was suggested. Involvement of Wnt activation into this process represents an initial submolecular change in ectopic endometrium. Finally, intensive expression of *CTNNB1 was* even in eutopic endometrium during menstruation observed.

**Fig. 1:**
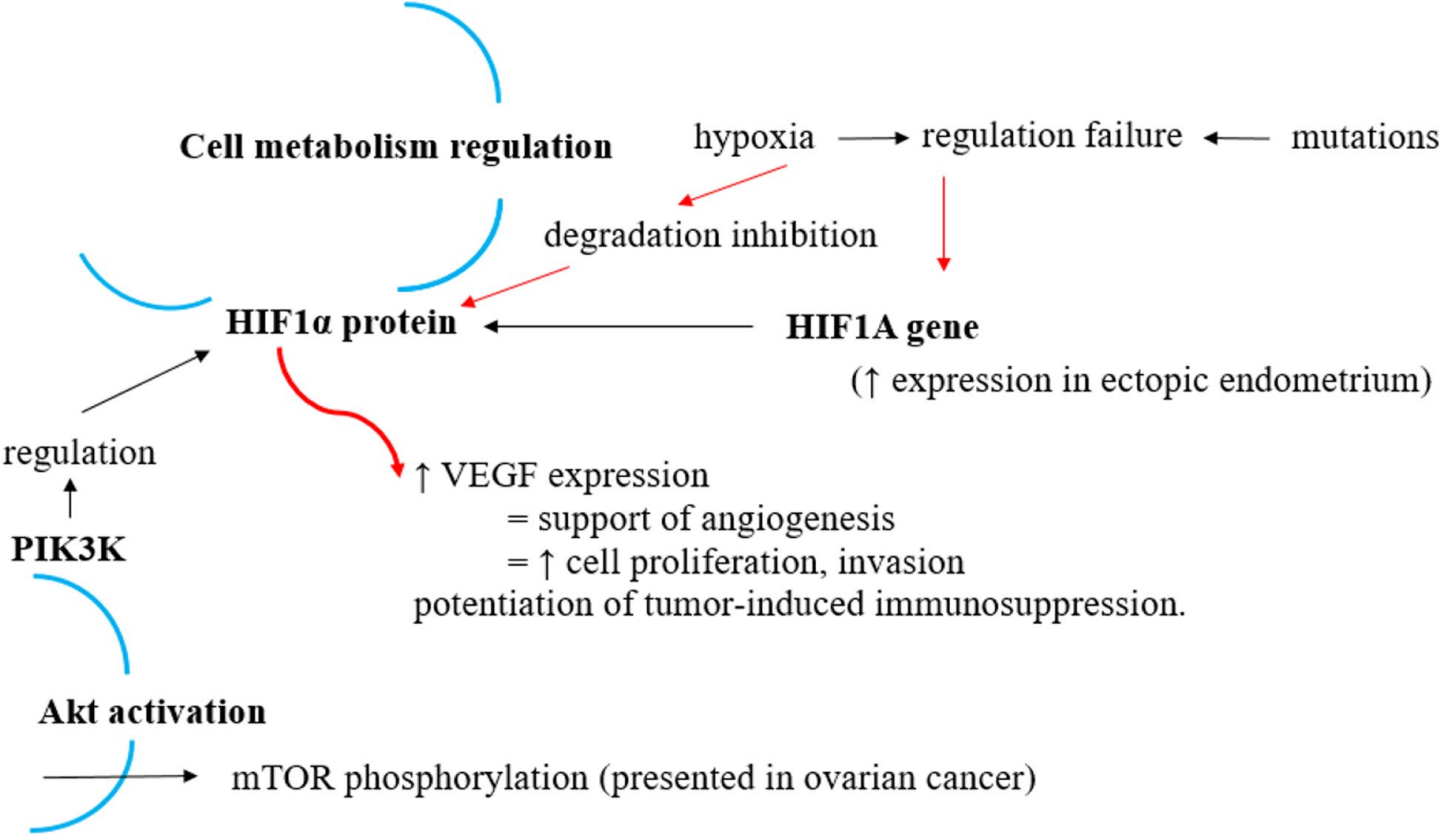
The role of HIF-1α in cell metabolism

**Fig. 2:**
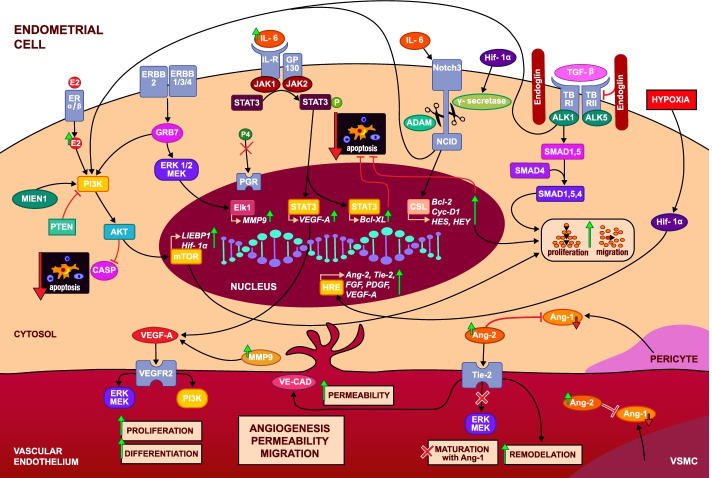
In a hypoxic environment, hydroxylation and degradation of HIF-1α are inhibited. Therefore, HIF-1α can dimerize, enter the nucleus and transcriptionally regulate the expression of its target genes through the transcription factor HRE. This way it regulates a wide range of pathophysiological processes including angiogenesis. The inflammatory and hypoxic microenvironment in the endometrium regulates the expression of several proteins such as receptors ERBB, NOTCH3 and TGF-β, which trigger a cascade of signalling pathways (JAK/STAT, SMAD and PI3K/AKT/mTOR) finally leading to increase in gene expression VEGF, PDGF, Bcl-XL, MMP9, Ang-2 and Tie-2. The result is culmination in proangiogenic transcriptional responses including proliferation and migration, and inhibition of apoptosis. Increased expression of Ang-2 which competitively binds Tie-2, inhibits Ang-1/Tie-2 signaling and negates its stabilizing effects. The destabilizing effect of Ang-2 on blood vessels and the proliferative and migratory effects of VEGF lead to vascular growth and angiogenesis. [eNOS = endothelial nitric oxide synthase, ERK = extracellular signal-regulated kinase, MAPK = mitogen-activated protein kinase, PDGF = platelet-derived growth factor, PGF = placental growth factor, PI3K = phosphatidylinositol-3-kinase, HRE = hypoxia responsive element, GRB7 = Human Growth Factor Receptor Bound Protein 7, NOTCH3 = Human Neurogenic Locus Notch Homolog Protein 3, PGR = Human progesterone receptor, MIEN1 = Migration And Invasion Enhancer 1, ERBB = the human epidermal growth factor receptor, JAK = Janus kinases, STAT = signal transducer and activator of transcription proteins, MMP9 = matrix metallopeptidase 9, CASP = cysteine-aspartic proteases, P4 = progesteron]

Protein hypoxia-inducible factor 1-alpha (HIF-1α) presents a subunit of transcription factor hypoxia-inducible factor 1 (HIF-1), encoded by gene *HIF1A.* This protein is involved in the processes of cell metabolism regulation, in particular its response to hypoxia (Fig. 1). Hypoxia represents a condition important for angiogenesis, physiologically seen in embryo or as the part of pathological process in tumour tissue. Hypoxia inhibits HIF-1α degradation leading to its translocation into cell nucleus and subsequent activation of the expression of various genes as well as vascular endothelial growth factor (VEGF). The result of these processes is pro-angiogenic potential of the tissue. Thus the main function of HIF-1α is regulation of VEGF, secondly it contributes to the potentiation of tumour-induced immunosuppression. HIF-1α regulation is also performed by PI3K which provides an activation of Akt signaling pathway. The PI3K/Akt pathway is an intracellular signaling pathway involved in several processes such as cell proliferation, apoptosis, angiogenesis or glucose metabolism. PI3K activation phosphorylates and activates Akt. Akt phosphorylates different substrates, including mTOR (mammalian target of rapamycin) which activation was in case of OC seen (Fig. 2). In this scenario the decrease in level of mTOR, HIF-1α and VEGF is the goal of the targeted therapy for OC. Many cancers show overactive Akt pathway resulting in apoptosis reduction or proliferation. Significantly higher expression of *HIF1A* gene was seen in ectopic endometrium comparing to normal endometrium [17].

The relationship between level of HIF-1α and CTNNB1 and the potential of endometriosis malignization was not studied yet.

The goal of the presented study was to review a malignant potential of the endometriosis by usage of three methods and to specify predictive factors of endometriosis progression into OC.

Conventional cytogenetics was used to reveal raw genetic changes in endometriosis patients. Histopathological analysis evaluated a presence of AE in endometriosis patients as well as presence of different type of endometriosis in EAOC patients. By the PCR examination of the mentioned genes we were trying to select a high risk patients with endometriosis where the probability of progression into cancer is significantly seen.

## Material and methods

Altogether 189 patients were included in the study. Prospectively conventional cytogenetics as well as PCR examination of the genes *HIF1A* and *CTNNB1* was performed in 60 patients with endometriosis. In addition, to compare results of PCR examination both genes were assessed in 50 healthy patients as well. Retrospective histopathological analysis was done in 79 patients. There were 60 patients with endometriosis and 19 patients with EAOC.

### Conventional cytogenetics

Basic genetic examination, conventional cytogenetics is performed by banding technique which creates a patterns of horizontal bands on the examined chromosomes. The most commonly used technique include G-band forming bright (euchromatin, active regions of chromosomes) and dark (heterochromatin, inactive genes) horizontal bands along chromosomes. By the examination structural and numerical aberration of the chromosomes can be seen. Cytogenetic examination can be performed from peripheral blood, fetal cells, but also from vital tissue.

Prospectively 60 reproductive age patients (20-56 years, average age 36.9 years) with histologically proven endometriosis were assessed by this method. In all the patients a peripheral blood was for the conventional cytogenetics used. The characteristics, i.e. surgical approach, perioperative finding, type of surgery as well as other findings are shown in Table 2. Peripheral venous blood (3-5 ml) was after harvesting transported into the laboratory till 60 min. The process of tissue preparation composed from three phases which is well known and has been described in detail:

1. Lymphocytes cultivation
2. Preparation of cytogenetic sections
3. Staining of cytogenetic sections

**Table 2:** Patients for cytogenetic and PCR examination

**PATIENTS**	60
**AVERAGE AGE**	36.9 years
**AVERAGE MENARCHE**	13.05 years
**NULLIGRAVIDA**	28 patients (47%)
**INFERTILITY**	Primary = 5 patients (8%), Secondary = 2 patients (3%), Without = 53 patients (89%)
**ONCOMARKER AVERAGE**	CA125 = 83.79 HE4, ROMA, CEA, CA 19-9 = normal finding
**SURGICAL APPROACH**	Laparoscopy = 36 (60%), Laparotomy = 20 (33%), Laparoscopy + laparotomy = 4 (7%)
**PERIOPERATIVE FINDING**	Endometrioma = 34 (57%), Peritoneal endometriosis = 6 (10%), Endometriosis of sacrouterine ligaments = 6 (10%), Endometrioma + peritoneal endometriosis = 10 (17%), Frozen pelvis = 4 (6%)
**TYPE OF SURGERY**	Extirpation of endometriomas = 30 (50%), Adnexectomy = 12 (20%), Extirpation of endometriotic lesion = 8 (13%), Biopsy of endometriotic lesion = 7 (12%), Hysterectomy = 3 (5%)

### Histopathological analysis

Altogether 79 patients were retrospectively examined in this section, i.e. 60 patients with endometriosis and 19 with EAOC.

During the years 2007-2014 from 178 OC patients were 19 patients categorized as EAOC. Inclusion criteria were histology of EOC or CCOC. The age of the patients was 43-68 years with average age 52.36 years. Five patients were presented with bulky tumor and average diameter more than 11 cm. Excepts in 3 patients the CA125 was examined preoperatively and the average value was 250 IU/ml. All the patients’ characteristics are listed in Table 3.

**Table 3:** Characteristics of EAOC patients for histopathological analysis

**PATIENTS**	19
**AVERAGE AGE**	52.36 years
**PRIMARY DIAGNOSIS**	Tumour adnex l. sin. = 12 patients Tumour adnex l. dx. = 7 patients
**ONCOMARKER AVERAGE**	CA125 = 250 IU/ml, CA 19-9 = 1048 IU/ml
**TYPE OF SURGERY**	Radical surgery = 14 patients, Hysterectomy with bilateral adnexectomy = 5 patients
**HISTOLOGY**	EOC = 12 (63.15%) patients, CCOC = 7 (36.85%) patients
**OTHER FINDINGS**	Nulligravida = 5, bulky tumour = 5

Retrospectively selected 60 patients with ovarian endometriosis were histopathologically analyzed. The average age of the included patients was 33.4 years (19-62 years). All the patients’ characteristics are listed in Table 4.

**Table 4:** Characteristics of endometriosis patients for histopathological analysis

**PATIENTS**	60
**AVERAGE AGE**	33.4 years
**PRIMARY DIAGNOSIS**	Tumour adnex l. dx. = 32 patients Tumour adnex l. sin. = 24 patients Bilateral tumour = 4 patients
**SURGICAL APPROACH**	Laparoscopy = 52 patients, laparotomy = 8 patients
**TYPE OF SURGERY**	Extirpation of endometrioma = 38 patients, adnexectomy = 19 patients, hysterectomy and bilateral adnexectomy = 3 patients
**OTHER FINDINGS**	Nulligravida = 13, CA125 elevation = 9, infertility = 4

The criteria for diagnosis of AE were already published [18] and this classification was used for definition of AE in selected patients. Thus the patients with endometriosis were classified either as a BE or AE. While in EAOC patients the possibilities of histopathological findings were as follows:

1. EAOC without endometriosis
2. EAOC with BE
3. EAOC with AE
4. EAOC with histologically proven gradual development from BE through BOC to EAOC.
5. To avoid a subjective evaluation all the slides in both groups were examined by one pathologist. The main specialization of the pathologist is focused to endometriosis and ovarian pathology.

### PCR analysis

A prospective PCR analysis of selected genes was done in 110 patients.

The experimental group (EG) consisted of 60 patients with histologically confirmed endometriosis where conventional cytogenetics was also performed (Table 2). The average age of included patients was 36.9 years (20-56 years) and the endometriosis patients were free of other diseases.

The control group (CG) was made up of 50 blood donor patients. The inclusion criteria were negative family history of oncological disease in last two generation, negative medical examination, blood findings within reference range including oncomarker CA125 and CEA. Every patient from CG had vaginal ultrasonography with negative result. The average age was 34.2 years (21-46 years).

The real time PCR method was used to investigate evidence of expression changes on mRNA levels. Four analyses of each gene, per person, in EG and CG were performed. Total RNA was isolated from peripheral whole venous blood using a *RNA blood Mini isolation kit* (Qiagen). Total RNA was quantified and purity was assessed using the Nanodrop® 3300 (Thermo Scientific). Reverse transcription from mRNA to cDNA was achieved using RevertAid Minus First Strand cDNA Synthesis Kit (Merck) with specific reverse primers for each gene. After the definition of reaction conditions for SensiMix™ SYBR® NoROX kit ran the amplification of the specific gene HIF1A and β-catenin for 30 cycles (94 °C 5 min, 94 °C 15 s., 60 °C 20 s. and 72 °C 25 s.), using appropriate primer sequences in the thermocycler *LightCycler*® 480 Instrument II (Roche Life Science). Normalization of the results was performed using housekeeping gene HGPRT and GAPDH. Numerical quantification of changes in expression levels was evaluated using the LightCycler® 480 Software, Version 1.5., where were confirmed Ct values corresponded with the midpoint of logarithmic amplification. Relative mRNA concentrations were calculated with respect to reference RNAs and inter-class fold changes computed using the 2^-ΔΔCt^ function.

In order to minimize the impact of variability in the experimental data, all samples were measured four times. For the statistical evaluation One-Way ANOVA Student–Newmann–Keuls test was used. Data is presented as mean percent ± SD. Statistical analysis was processed by the program GraphPad INSTAT.

## Results

### Conventional cytogenetics

From 60 endometriosis patients were 55 identified with normal karyotype 46,XX. 5 patients (8.3%) showed cytogenetic deviation from the physiological finding.

Three of them were concluded as heteromorphisms – variants of human karyotypes without affecting of patients’ phenotype. Such non-aberrant karyotype changes affect those parts of the chromosomes whose length or molecular structure varies within a population. All the deviations were detected in acrocentric chromosomes – group D (chromosomes 13, 14, 15) and group G (chromosomes 21, 22):

1. chromosome 14 satellite duplication (46,XX, 14pss)
2. absence of chromosome 13 satellite (46,XX, 13 ps-)
3. the association between group D and G chromosomes – chromosomal configuration with potential of chromosomes withdrawal during next division and forming of numeric aberration.

In the rest of pathological findings – in two patients, the numerical abnormality with manifestation in the mosaic form was confirmed:

1. 45,X(5)/46,XX(45) – in five mitosis the karyotype 45,X was seen. The rest of mitosis was with karyotype 46,XX. In this case Turner mosaic was confirmed.
2. 46,XX,+mar(2)/46,XX(98) – in two mitoses a marker chromosome from group C was seen. The case was concluded as the mosaic form of X chromosome trisomy (superfemale) with normal phenotype.

By comparison of the results with clinical characteristics of the patients the association was not observed. The patients containing a cytogenetic deviations were diagnosed with not extensive endometriosis. In three of them an ovarian endometrioma and in two of them endometriosis of sacrouterine ligament was seen. Complete resections were in all patients performed.

### Histopathological analysis

Altogether 19 (10.67%) of 178 OC patients diagnosed between 2007 and 2014 were confirmed as EAOC. From all EAOC patients 12 of them (63.15%) were EOC and 7 (36.85%) CCOC.

Overall in 19 EAOC patients the endometriosis was not seen in 6 (31.57%) patients. In 3 patients (15.78%) the tissue of BE was confirmed while AE in 4 EAOC patients (21.08%). In other 6 patients the transition from BE through BOC structures to carcinoma tissue was identified.

When we checked for EOC (12 patients), 3 (25%) of them were without endometriosis, 2 (16.66%) with BE, 3 (25%) with AE and finally 4 (33.34%) EOC patients had transition from BE to cancer through BOC in their histology.

From seven CCOC patients, 3 (42.85%) were free of endometriosis, both BE as well as AE was confirmed in 1 patient (14.28%). Two (28.59%) CCOC patients had except carcinoma cells also BE and BOC in histology finding.

**Fig. 3:**
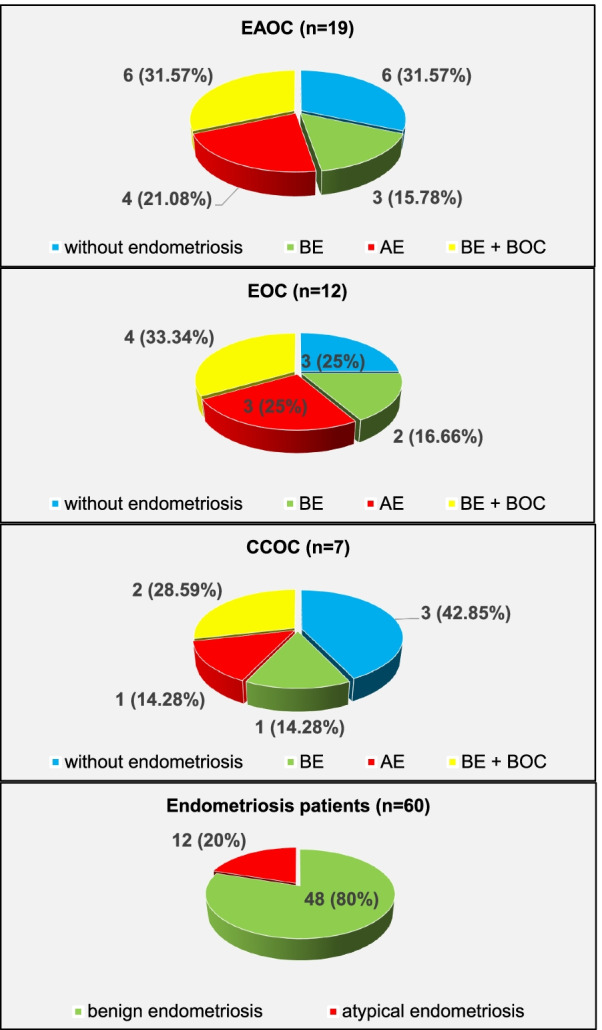
Histopathological analysis of EAOC, EOC, CCOC and endometriosis patients

From 60 ovarian endometriosis patients were 12 (20%) patients classified with AE while the rest 48 (80%) showed only BE finding (Fig. 3).

### PCR analysis

The analysis of *HIF1A* and *CTNNB1* (mRNA level) in CG patients (*n* = 50) represented a reference value compared with the findings in the EG patients (*n* = 60).

The patients in EG were regarding the level of gene transcription divided into two subgroups. Transcriptionally incipient endometriosis (TIE), with 26 patients showed increased level of gene mRNA for both, *HIF1A* and *CTNNB1* compared to CG however significantly lower than in second group. This group - transcriptionally evident endometriosis (TEE), contained 34 patients from EG where the level of mRNA genes (*HIF1A* and *CTNNB1*) were significantly higher compared to the both, CG as well as TIE (Table 5).

**Table 5:** PCR gene analysis in control and experimental group

Group/subgroup	Patients	Gene expressions (mRNA level)	
		*HIF1A*	*CTNNB1* (*β-catenin*)
**CG**	*n* = 50	minimum – 0.98 maximum – 1.081 **median – 1.00**	minimum – 0.984 maximum – 1.032 **median – 1.00**
**EG/TIE**	*n* = 26	minimum – 1.16 maximum – 1.299 **median – 1.21**	minimum – 1.086 maximum – 1.126 **median – 1.094**
**EG/TEE**	*n* = 34	minimum – 1.484 maximum – 1.613 **median – 1.563**	minimum – 1.328 maximum – 1.567 **median – 1.499**

**Fig. 4:**
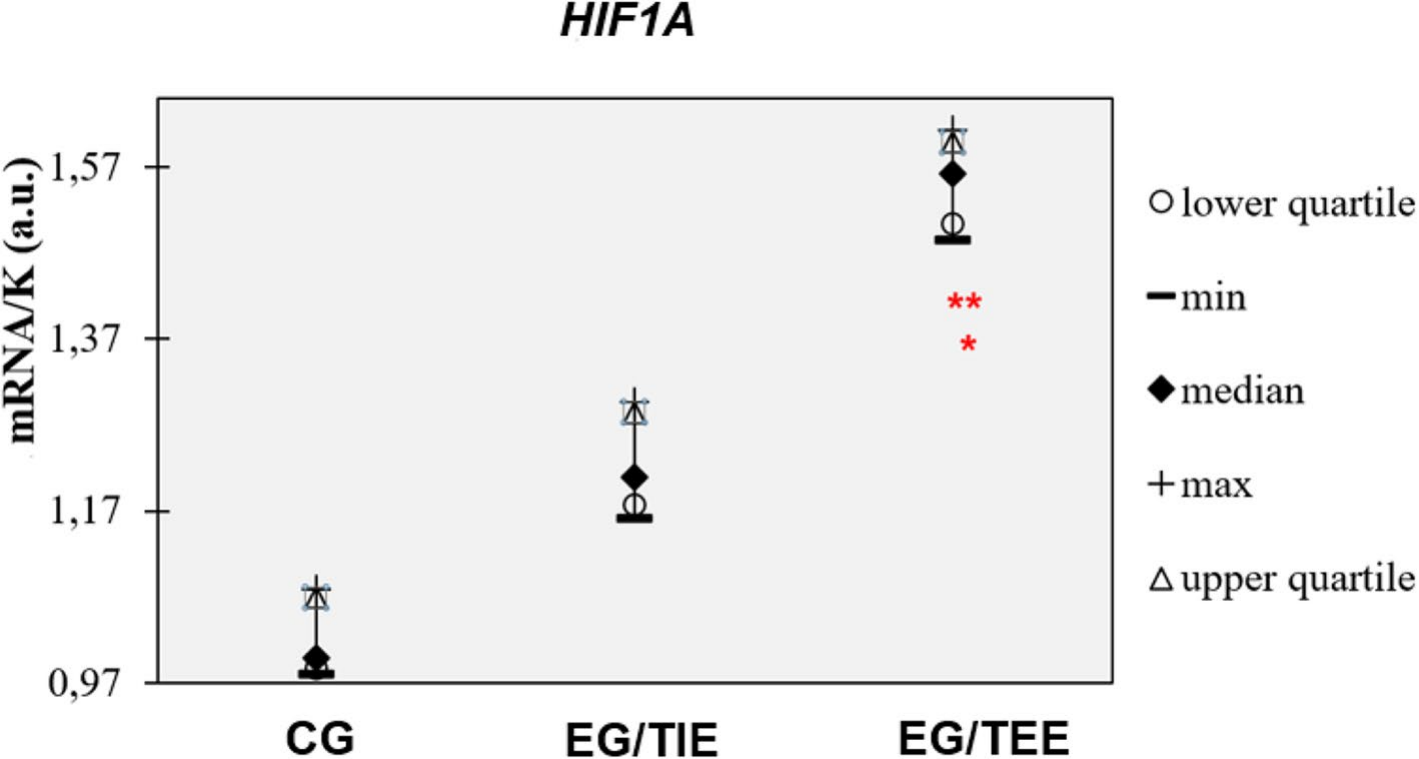
*HIF1A* expressions in CG, EG/TIE and EG/TEE. *p <0.05 EG/TEE vs. EG/TIE, ** *p* <0.01 EG/TEE vs. CG

*HIF1A* expressions in TIE group (median 1.21) were increased without significance when compared to CG (median 1.00). However TEE group expressions (median 1.563) were significantly higher compared to CG patients (*p* < 0.01) as well as TIE patients (*p* < 0.05) (Fig. 4).

**Fig. 5:**
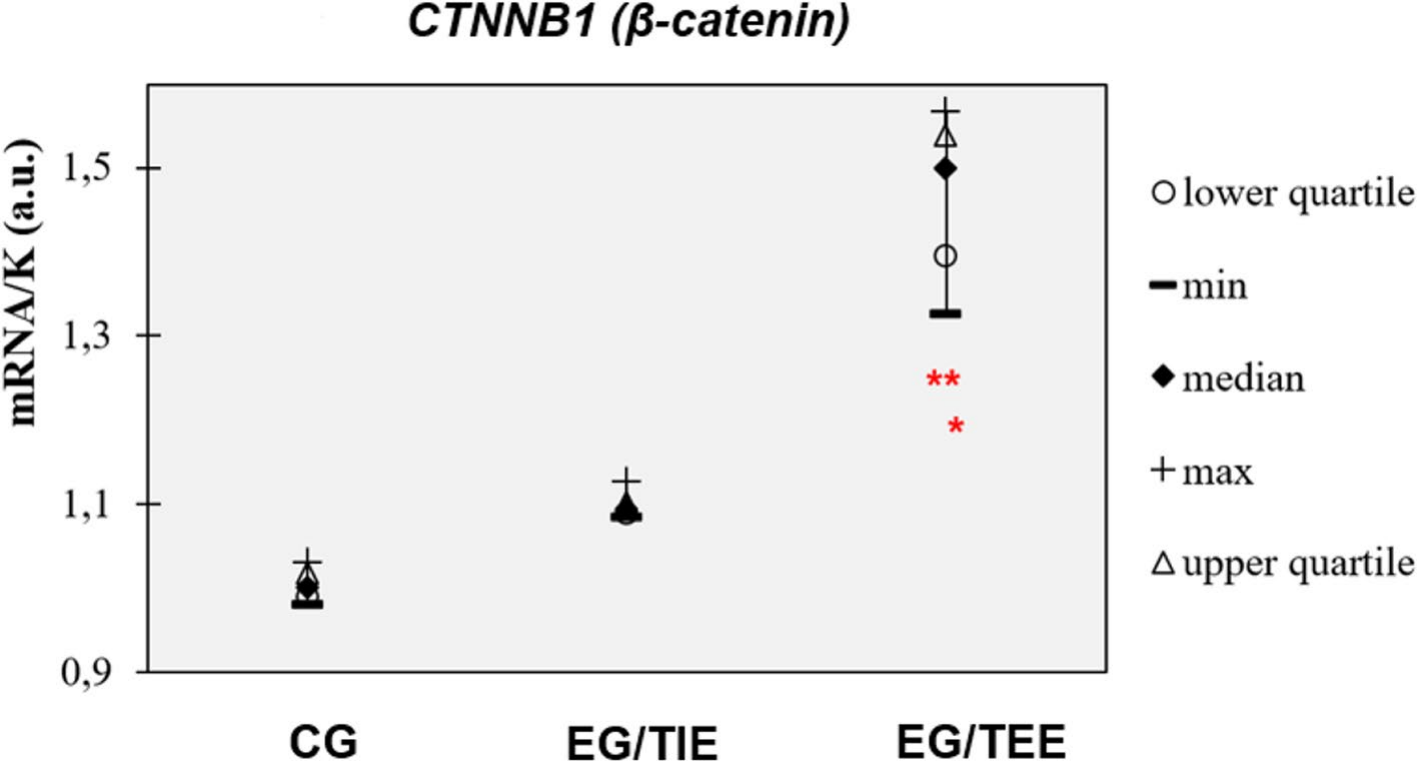
*CTNNB1 (β-catenin)* expressions in CG, EG/TIE and EG/TEE. *p <0.05 EG/TEE vs. EG/TIE, ** *p* <0.01 EG/TEE vs. CG

The similar phenomenon was also in the assessment of *CTNNB1* seen. Into TIE group (median 1.094) were selected patients with increased expression comparing to CG (median 1.00) but without statistical significance. While significantly increased expressions compared to CG (*p* < 0.01) as well as TIE (*p* < 0.05) were seen in the rest of the patients included into TEE group (median 1.499) (Fig. 5).

## Discussion

The endometriosis was solidly confirmed as the precursor of certain portion of EAOC. Relatively low incidence of endometriosis overthrow makes difficult to define the predictive factors of this process. Tubo-ovarian junction plays a crucial role in process of malignant ovarian transformation even in the case of endometriosis. Inflamed stroma together with mutated alleles of the epithelial component is incorporated into CIC where due to the favorable environment a malignant transformation of endometriosis occurs [4]. The same microscopic changes are also in deep infiltrated endometriosis seen however malignant overthrow is rare [5]. In this direction ovarian endometriomas have due to their close connection to the ovaries the highest probability to progress into EAOC. The DNA alterations in endometriomas originate from permanent oxidative stress inside. Significantly higher iron ions concentration in endometriotic cysts was confirmed when compare to non-endometriotic cyst. Higher iron concentration was also in CCOC tissue seen, although the level did not reach the amount measured in endometriotic cysts [19]. Following this parameters the endometriosis patients included in presented study for histopathological examination were only with endometriomas. While cytogenetic and experimental PCR study was done in the patients with different types of endometriosis.

The development of genetics in the last decades has significantly influenced the focus of the study of various diseases, including endometriosis. Until 1997, conventional cytogenetics was predominant in genetic studies in endometriosis patients. Cytogenetics examinations over the last 20 years yielded discrepant results [20, 21], although the common feature can be defined as the absence of gross chromosome abnormalities. Conventional cytogenetics did not confirm typical chromosomal structural changes which are common for patient with endometriosis [22, 23]. Opposite conclusions were obtained by the studies applying fluorescence in situ hybridization (FISH) and comparative genomic hybridization (CGH). Mainly monosomy, structural aberration or presence of recurrent gene copies increasing clonality potential were confirmed and predominantly chromosomes 1, 16 and 17 were affected [24, 25]. It is well known fact that somatic mutations of chromosome 17 are often seen in process of ovarian carcinogenesis [26]. Three patients of presented study with heteromorphisms – variants of human karyotypes had the aberrations without affecting of patients’ phenotype. Although the changes are benign the karyotype can be instable leading to the problems in meiotic division. All the changes were seen in the group of chromosomes D and G. The association of group D and G chromosomes can affect the division leading to numerical aberration. From the presented results it is possible to support the conclusion that usage of conventional cytogenetics did not reveal structural abnormalities in karyotype of endometriosis patients.

**Fig. 6:**
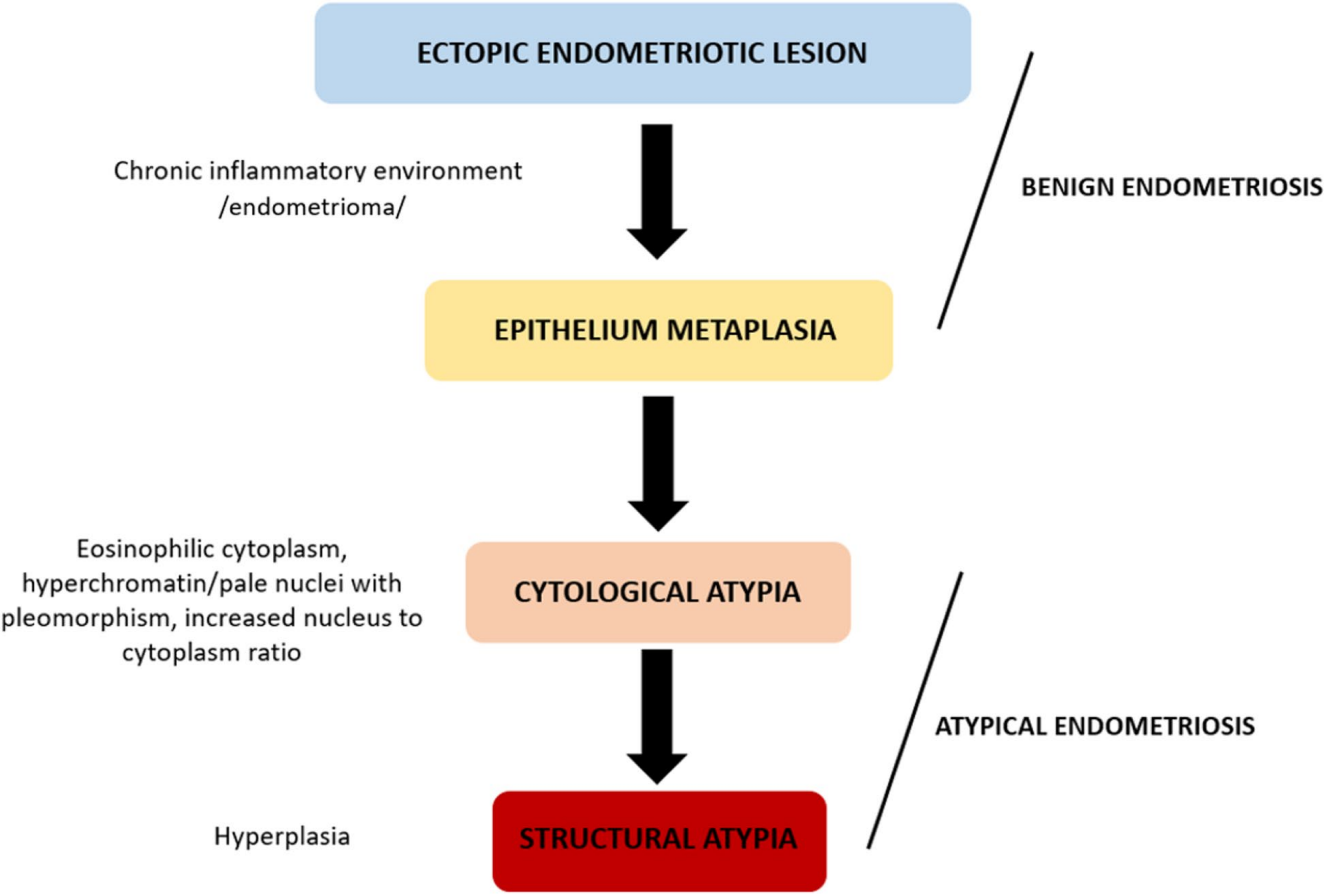
The development of atypical endometriosis

The switchover between endometriosis and ovarian cancer through carcinogenesis presupposes common morphological coherence. An extensive effort has been made to define morphological precursor of endometriosis overthrow. The initial concept was presented by Sampson in 1925 [27]. Later on the criteria have been extended by Scott in 1953 [28]. The group of borderline carcinomas defined WHO in 1971. Despite of logical context of the development of BE through mild cell atypia (cytological atypia) as the consequence of the inflammatory process to structural atypia (hyperplasia) in cases of AE, the criteria for definition of AE remain controversial and widely discussed. The chronic inflammatory changes in endometriomas lead to metaplastic reaction of the epithelium. The changes are diagnosed as benign and they have no clinical impact. Most probably they represent an early changes starting the process of AE formation. Nowadays widely accepted criteria of AE include structural changes as mentioned earlier (Fig. 6). Generally, not specified endometriosis patients, with all types of endometriosis, the incidence of AE is reported to be 8-10%. Higher incidence of AE in endometriosis patients was seen in presented study. 20% of studied histology showed signs of atypia. Reported increased incidence may be caused by types of patients. Due to the close connection with the ovaries only endometriomas were studied and not different types of endometriosis. Detailed analysis of patients with AE confirmed the patients with long-term history of disease, advanced finding during surgery and higher age in AE patients (39.8) when compare to patients with BE (31.8). The patients with long-term history as well as large endometriomas (> 9 cm) have been already confirmed at higher risk for progression and are indicated to stricter observation [29, 30].

The frequency of endometriosis overthrow is reported to be 0.3-0.8% [30]. The appearance of AE increases in case of OC patients. Similar phenomenon confirmed Niguez Sevilla with colleagues as well. 8.8% of AE was seen in cancer free patients however in EAOC patients the AE was confirmed in 34.6% [10]. In our study AE was seen in 21.08% of EAOC patients and most of them were with EOC histology (25%), lesser in CCOC patients (14.28%). Slightly different results of AE appearance in EOC and CCOC patients were published. 23% of EOC patients and 36% of CCOC patients were with a presence of AE in their histology [19, 30]. Generally the endometriosis was seen in 68.43% EAOC patients including benign, atypical type or benign endometriosis with BOC structures. EOC patients revealed 75% cases with endometriosis while CCOC patients 57.14%.

After 1953 many authors described in EAOC the transition starting from benign endometriosis to AE and BOC. When we checked this group, 31.57% EOAC patients in histology showed endometriosis with BOC, in EOC 33.34% and in CCOC 28.59%. This correspondences with literature. EAOC may have in their histology BE, AE or BE with BOC but also it can be without endometriosis structures [12]. The stepwise development containing endometriosis as well as structures of BOC was in 25% EAOC patients confirmed [10]. Following the results, the presence of AE in histology represents a significant risk factor and predisposes to more precise observation of the patient.

Wei with colleagues observed the presence of endometriosis in EAOC patients in younger women, average 45 years [30]. This average decreases mainly the CCOC patients while EOC patients tend to incease it. When we checked our results the average age of CCOC patients was 49.8 years and EOC 53.8 years. Surprisingly the average age in both groups, i.e. EOC and CCOC was lower in endometriosis free patients. The EOC patients without endometriosis were with average age 52.6 years while in patients with endometriosis the average age was 54.2 years. Same phenomenon was in CCOC patients seen. Endometriosis free patients were 47 years old in average while CCOC patients with endometriosis were 52 years old. The patients with EOC histology were captured in earlier stages with more common stepwise development form BE to BOC. On the other hand CCOC patients were diagnosed with more advanced stage with metastases in abdominal cavity.

The role of β-catenin protein in processes of carcinogenesis have been confirmed in many cancers. The mutations of *CTNNB1* gene were seen in pulmonary cancer, breast cancer, colorectal cancer, but also in endometrial and ovarian cancer, including EAOC [15]. Immunohistochemical positivity was confirmed in 61.2% EOC patients but both types of patients, i.e. endometriosis free and EOC with endometriosis showed this result [31]. High incidence, 90%, of *CTNNB1* mutation was also in endometrioid borderline carcinoma confirmed [32]. These findings suggest that the *CTNNB1* mutations may represent an early change in the process of malignization of some ovarian tumors.

The activation of Wnt signaling pathway which also includes a *CTNNB1* mutation is connected with the initiation of surrounding fibrotization [13], potentiation of proliferation, implantation or invasion [14]. This process was also in endometriosis confirmed [16]. The extent of the consequences can therefore be logically confronted with the extent of the protein defect.

Increased expression of *CTNNB1* was seen in presented endometriosis patients of EG when compare to CG. When insignificant increase was detected the patients were closed in TIE group. In the second subgroup of EG, i.e. TEE only significantly increased *CTNNB1* expressions were included. When we checked for clinical characteristics of the patients in TEE mainly advanced stage endometriosis patients were seen. Due to the stage of the disease they were often confronted with suboptimal surgery and up to 53% of them had macroscopic residual disease after surgery (Table 6). Taking into account both subgroups we can conclude that *CTNNB1* expression correlates with the endometriosis extent in patients.

**Table 6:** Patients’ characteristics in TIE and TEE

Group	Peroperative finding No. patients (%)		Type of surgery No. patients (%)		Residual disease No. patients (%)	
	Unextensive	Extensive	Optimal	Suboptimal	NEGAT.	POZIT.
**TIE (*n*** **= 26)**	20 (76.9%)	6 (23.1%)	25 (96.1%)	1 (3.9%)	22 (84.6%)	4 (15.4%)
**TEE (*n*** **= 34)**	14 (41.2%)	20 (58.8%)	22 (64.7%)	12 (35.3%)	22 (47.05%)	18 (52.9%)

Protein HIF-1 is included in the processes of cell metabolism regulation. Hypoxia leads to decrease in HIF-1α degradation resulting in pro-angiogenic potential as well as potentiation of tumor induced immunosuppression. This situations were in tissues of different carcinomas and endometriosis confirmed. Whereas in endometriomas an angiogenesis is more prominent in the outer capsule of the cyst comparing to inner part [17]. The inner environment of the tumours is characterized by low pH and low oxygen level due to the inadequate circulation. The fast proliferation of the tumor cells leads to the chronic ischemia mainly in central part of the tumours. The result of oxygen depletion is up-regulation of different genes including *HIF1A*. Finally the VEGF and HIF-1α elevation positively correlate with worse prognosis of OC patients [33]. Therefore the antibodies against VEGF are used as target therapy in clinical practice. The development of *HIF1A* expressions showed a similar results. In the group TIE only insignificant *HIF1A* expressions increase were included. They showed increasing about 9.4% when compared to CG. The significant elevation comparing to CG as well as TIE was in the rest of patients seen. They were selected into TEE group with 49.9% elevation comparing to CG. The results are comparable with the previous studies [17, 34] although not every patient showed significant increasing. In the analysis of clinical characteristic of the patients was in the TEE group apparently higher amount of advanced stage patients seen. Also patients with suboptimal surgery or macroscopic residual disease were mainly in this subgroup detected (Table 6).

Significant increase in mRNA of both genes *HIF1A* and *CTNNB1* was in TEE seen. Those patients revealed mainly extensive preoperative finding. Bilateral endometrioma, frozen pelvis and peritoneal endometriosis in coincidence with endometriomas were defined as extensive extent. While unilateral endometrioma as well as isolated endometriosis of sacrouterine ligament were defined as unextensive finding.

## Conclusion

Endometriosis represents a heterogeneous disease from many aspects. The need for the selection of the patients is appropriate and uniform management is obsolete at present. In terms of possibility of endometriosis overthrow the cognition of predictive factors is the basis of the issue. Endometriomas due to their close relationship to ovarian microenvironment are more susceptible to malignant overthrow. Tubo-ovarian junction plays important role in ovarian carcinogenesis, including those containing endometriosis. Specific structural chromosome abnormalities in endometriosis patients predicting its malignant overthrow are not detectable by the conventional cytogenetics. By applying the histopathological criteria defining AE a risk group of endometriosis patients with need for more precise observation can be selected. The patients with long-term history of endometriosis, advanced stage and big endometriomas should be under precise observation. EAOC is more common with endometriosis. EOC are usually less aggressive, slowly progressing and often with detectable histological transition from precursor to invasive carcinoma. Increased expression of mentioned genes is most probably related to the disease progression and characterizes early stages of progression. Determination of genes transcription can help to select a risk group of patients, although other studies are needed.

## Data Availability

The primary data for this study is available from the authors on direct request.

## Abbreviations

AE: Atypical endometriosis
BE: Benign endometriosis
BOC: Borderline carcinoma
CCOC: Clear cell ovarian cancer
CG: Control group
CGH: Comparative genomic hybridization
CIC: Cortical inclusion cyst
EAOC: Endometriosis associated ovarian cancer
EG: Experimental group
EOC: Endometrioid ovarian cancer
FISH: Fluorescence in situ hybridization
HGSOC: High grade serous ovarian cancer
LGSOC: Low grade serous ovarian cancer
OC: Ovarian cancer
OSE: Ovarian surface epithelium
PTH: Papillary tubal hyperplasia
STIC: Serous tubal intraepithelial carcinoma
TEE: Transcriptionally evident endometriosis
TIE: Transcriptionally incipient endometriosis
VEGF: Vascular endothelial growth factor
